# Modulation of Innate Antiviral Immune Response by Porcine Enteric Coronavirus

**DOI:** 10.3389/fmicb.2022.845137

**Published:** 2022-02-14

**Authors:** Kunli Zhang, Sen Lin, Jianhao Li, Shoulong Deng, Jianfeng Zhang, Sutian Wang

**Affiliations:** ^1^State Key Laboratory of Livestock and Poultry Breeding, Guangdong Key Laboratory of Animal Breeding and Nutrition, Institute of Animal Science, Guangdong Academy of Agricultural Sciences, Guangzhou, China; ^2^Institute of Animal Health, Guangdong Academy of Agricultural Sciences, Key Laboratory of Livestock Disease Prevention of Guangdong Province, Scientific Observation and Experiment Station of Veterinary Drugs and Diagnostic Techniques of Guangdong Province, Ministry of Agriculture and Rural Affairs, Guangzhou, China; ^3^Sericultural & Agri-Food Research Institute, Guangdong Academy of Agricultural Sciences, Guangzhou, China; ^4^Institute of Laboratory Animal Sciences, Chinese Academy of Medical Sciences and Comparative Medicine Center, Peking Union Medical College, Beijing, China; ^5^Maoming Branch, Guangdong Laboratory for Lingnan Modern Agriculture, Guangdong, China

**Keywords:** porcine enteric coronaviruses, innate immunity response, immune evasion, PEDV, PDCoV, TGEV, SADS-CoV

## Abstract

Host’s innate immunity is the front-line defense against viral infections, but some viruses have evolved multiple strategies for evasion of antiviral innate immunity. The porcine enteric coronaviruses (PECs) consist of porcine epidemic diarrhea virus (PEDV), porcine deltacoronavirus (PDCoV), transmissible gastroenteritis coronavirus (TGEV), and swine acute diarrhea syndrome-coronavirus (SADS-CoV), which cause lethal diarrhea in neonatal pigs and threaten the swine industry worldwide. PECs interact with host cells to inhibit and evade innate antiviral immune responses like other coronaviruses. Moreover, the immune escape of porcine enteric coronaviruses is the key pathogenic mechanism causing infection. Here, we review the most recent advances in the interactions between viral and host’s factors, focusing on the mechanisms by which viral components antagonize interferon (IFN)-mediated innate antiviral immune responses, trying to shed light on new targets and strategies effective for controlling and eliminating porcine enteric coronaviruses.

## Introduction

As the largest positive-sense RNA viruses that exist widely in nature, coronaviruses have genetic diversity and host diversity. Specific coronavirus populations have been found in humans, mice, bats, pigs, chickens, cows, cats, dogs, and many other animals, some of which are zoonotic and pose serious threats to human health and livestock safety. Due to the mild clinical manifestations after infection, coronaviruses have long been ignored by people. However, the outbreak of Severe Acute Respiratory Syndromes (SARS) in 2003 caused a total of 8,000 cases of infection worldwide, including 774 deaths, with a mortality rate of about 10% (Drosten et al., 2003). Even worse, the worldwide spread of the Severe Acute Respiratory Syndrome Coronavirus 2 (SARS-CoV-2) since 2019 caused hundreds of millions of infections and millions of deaths. Its high infectivity and transmission speed and the lack of specific medicines and vaccines caused a worldwide panic. In the past few decades, with the continuous development of the breeding mode, various coronavirus, including porcine epidemic diarrhea virus (PEDV) and infectious chicken bronchial virus, have been world-widely prevalent and pose great challenges to the health and safety of the breeding industry. Some animals have been proved to carry coronaviruses that can spread from one species to another. A most recent research has revealed a surprising result showing that porcine deltacoronavirus (PDCoV) strains exist in plasma samples of three Haitian children with acute undifferentiated febrile illness (Lednicky et al., 2021). Therefore, systematic analysis of animal coronavirus, especially porcine enteric coronavirus (PEC), appeared to be extremely necessary.

Interferons (IFNs) are key components of the host’s antiviral innate immunity. IFNs are consisted of type I IFNs, type II IFNs, and type III IFNs. Type I IFN is a non-glycosylated protein composed of 165–300 amino acids. Almost all cells can produce type I IFNs when pattern recognition receptors (PRRs) recognize the microbial pathogen-associated molecular patterns (PAMPs). IFN-I binds to type IFN-I receptor (IFNAR) to induce a powerful antiviral defense program involving hundreds of interferon-stimulated genes (ISGs) by activating the JAK–STAT pathway. Furthermore, ISGs are capable of interfering with every step of viral replication (Schoggins and Rice, 2011). Like IFN-I, type III IFNs bind to the type III IFN receptor (IFNLR) and share the same pathway to induce a similar antiviral transcriptional program (Kotenko et al., 2019). It is now well-recognized that the IFN-λ-based antiviral system plays a major role in the antiviral protection of epithelial barriers. Due to the different expression of receptors, IFN-I signaling leads to a more rapid induction and decline of ISG expression. In contrast, IFN-III signaling induces the expression of ISGs in a more sustained way (Lazear et al., 2019). IFNs establish the cellular state of viral resistance and activate the adaptive immune responses to viruses. However, some viruses have evolved quite complicated mechanisms to escape immune recognition and antagonize the effects of IFNs and ISGs. The mechanisms by which different components of these viruses antagonize immune responses are also different. In the present review, the characteristics of PEC biology are elucidated, the mechanisms by which viruses antagonize immune responses are illustrated, and finally, the potential targets and strategies effective for controlling and eliminating porcine enteric coronaviruses are discussed.

## Overview of Porcine Enteric Coronaviruses

In 2019, the International Committee on Taxonomy of Viruses divided the Coronaviridae into Letovirinae and Orthocoronavirinae, containing five genuses: Alphacoronavirus, Betacoronavirus, Gammacoronavirus, Deltacoronavirus, and Alphaletovires. To date, there are six known swine coronaviruses, including four alphacoronavirus, one betacoronavirus, and one deltacoronavirus. Transmissible gastroenteritis coronavirus (TGEV), porcine respiratory coronavirus (PRCV), PEDV, and swine acute diarrhea syndrome-coronavirus (SADS-CoV) belong to the alphacoronavirus. Porcine hemagglutinating encephalomyelitis virus (PHEV) belongs to the betacoronavirus, and PDCoV belongs to the deltacoronavirus. Furthermore, the evolutionary genetic analysis suggested that PEDV and SADS-CoV were thought to originate from the bat CoVs and PDCoV from a sparrow CoV (Zhou et al., 2018; Wang et al., 2019), which suggested coronaviruses could spread from species to species. PEDV, PDCoV, and TGEV SADS-CoV can cause gastrointestinal infections and similar characteristics (Table 1). These porcine enteric coronaviruses mainly affect the digestive tract of piglets, and the clinical symptoms include weight loss, lethargy, vomiting, anorexia, watery diarrhea, and even death. The pathological features were necrosis and shedding of intestinal cells and intestinal villi injury (Jung et al., 2014; Pan et al., 2017; Suzuki et al., 2018; Xia et al., 2018). PEDV was first reported in the United Kingdom in the 1970s but was not found in the United States until 2013 (Wood, 1977; Stevenson et al., 2013). In the short time that followed, PEDV spread worldwide, with high morbidity and mortality rates, and caused huge economic losses to the global pig industry (Jung et al., 2015). The incubation period of the virus is generally 5–8 days. In addition, PEDV can infect pigs of all ages, but the severity and mortality of infected pigs are inversely proportional to pigs. The morbidity and mortality of suckling piglets within 7 days were up to 100% (Li et al., 2020). So far, PEDV has only been found to infect pigs and has no impact on public health.

**Table 1:** Characteristics of porcine enteric coronaviruses.

Viruses (Genera)	Year of emergence	Mortality in neonatal piglets	Pathogenicity for other species	Clinical symptoms
PEDV (Alphacoronavirus)	1970s	Almost 100%	No report	Vomiting, watery diarrhea, dehydration, and weight loss
PDCoV (Deltacoronavirus)	2009	50%–100%	Humans, Calves, chickens, and turkeys	Vomiting, watery diarrhea, dehydration, and weight loss
TGEV (Alphacoronavirus)	1946	Up to 100%	No report	Vomiting, watery diarrhea, dehydration, weight loss, and abortion
SADS-CoV (Alphacoronavirus)	2016	More than 90 in pigs ≤ 5 days of age	No report	Acute diarrhea, acute vomiting, and acute death

Since 2012, PDCoV has been detected in several countries, including China, the United States, Japan, and Canada (Woo et al., 2012; Wang et al., 2014b; Ajayi et al., 2018). PDCoV can cause diarrhea of piglets in different degrees and PDCoV disease, and the incidence and mortality of PDCOV disease in suckling piglets are about 50%–100%. The ability to spread across species is the most obvious feature of coronavirus. Researchers believed that PDCoV could only infect chickens and calves but not humans for a long time. Furthermore, the virus does not cause serious health problems in these animals (Jung et al., 2017; Liang et al., 2019; Boley et al., 2020). However, a research team has identified porcine deltacoronavirus strains in plasma samples of three Haitian children with acute undifferentiated febrile illness (Lednicky et al., 2021). This discovery makes us aware that these porcine coronaviruses may cause threats to public health.

Transmissible gastroenteritis coronavirus was first reported in the United States in 1946 and then broke out worldwide (Doyle and Hutchings, 1946; Kim et al., 2000). TGEV infection mainly causes infectious gastroenteritis, which leads to vomiting, watery diarrhea, and even death in piglets (Garwes, 1988). TGEV infection is fatal to piglets born less than 1 week, since the mortality rate can reach high up to 100%. Although the mortality rate of infected pigs over 2 weeks old is low, their growth and development are slow, which can cause economic losses to the pig breeding industry (Saif, 1999; Penzes et al., 2001). Significantly, TGEV is a highly contagious disease with a short incubation period (usually 1–3 days) and can quickly affect the entire pig population (Liu and Wang, 2021). Pigs are the only host of TGEV, and no human infection has been reported to date. SADS-CoV was first reported in the southeast of China in 2016, which is the sixth porcine coronavirus identified so far. The SADS-CoV infection leads to acute diarrhea, acute vomiting, and even acute death in piglets, and the mortality rate of virus infection in piglets within 5 days of age is more than 90% (Zhou et al., 2018). Tests on 35 people who had close contact with infected pigs found no evidence of human infection, suggesting the virus may not be capable of transmitting to humans.

## The Genome Structure and Function of Porcine Enteric Coronaviruses

The PEDV genome is about 28 kb in length and consists of 3′, 5′ untranslated regions (UTR) and seven open reading frames (ORFs). The ORF sequence is ORF1a, ORF1b, spike protein (S), ORF3, envelope protein (E), membrane protein (M), and nucleoprotein (N; Kocherhans et al., 2001). ORF1 occupies two-thirds of the length of the genome at the end of the 5′ UTR and encodes two proteins (pp1a and pp1ab), which can be hydrolyzed by papain-like protease and serine type 3C-like protease to non-structural proteins (NSP) 1α, NSP1β, and NSP3-16. And then, these NSPs participate in virus replication, transcription, translation, and viral protein processing (Kadoi et al., 2002; Huang et al., 2013). The S protein of PEDV consists of S1 (1-789aa) and S2 (790-1383aa) subunits. The S1 helps PEDV bind to host receptors, and S2 induces membrane fusion and PEDV invasion (Liu et al., 2015). In addition, PEDV S protein also induces neutralizing antibodies in the host body (Song and Park, 2012). PEDV ORF3 sits between the S and E genes and encodes ORF3 protein, which plays an essential role in virulence (Park et al., 2011). The E gene of PEDV is only 231 nt in length and encodes E protein, which is essential for virus assembly and budding (Brian and Baric, 2005). The M gene of PEDV is 681 nt in length and can encode M protein (226aa), which is a transmembrane protein on the viral envelope (Narayanan et al., 2000). It has been reported that M protein is involved in virion assembly, budding, and host innate immune induction (Utiger et al., 1995). The PEDV N protein is a highly conserved protein that consists of 441aa and is involved in the survival of the virus (Wang et al., 2020c).

**Figure 1:**
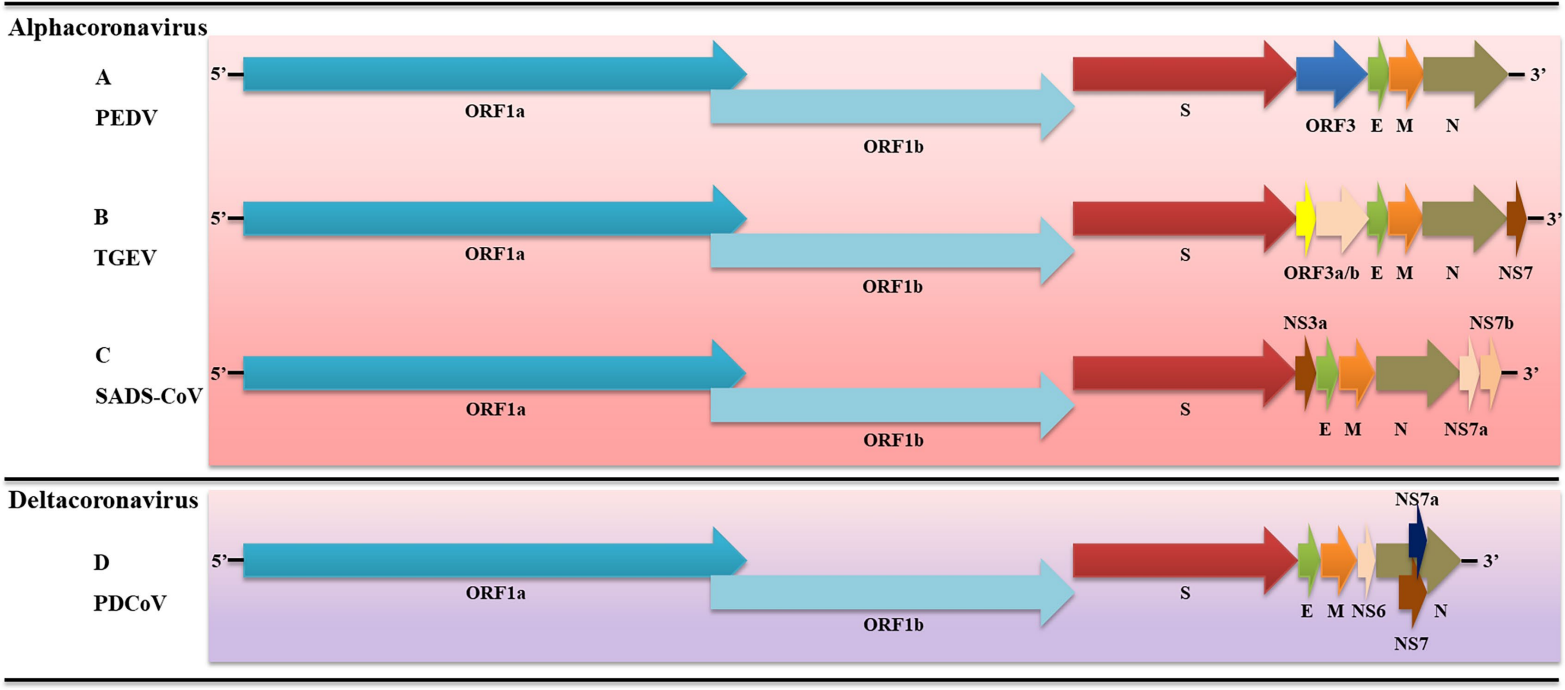
Schematic diagram of porcine enteric coronaviruses (PECs). **(A)** Genome structure of PDEV; **(B)** Genome structure of transmissible gastroenteritis coronavirus (TGEV); **(C)** Genome structure of swine acute diarrhea syndrome-coronavirus (SADS-CoV); **(D)** Genome structure of porcine deltacoronavirus (PDCoV). S, spike; E, envelope; M, membrane; N, nucleoprotein; and Ns, accessory genes.

The TGEV genome is about 28.5 kb in length, consisting of 3′, 5′ UTR, and seven open reading frames. The gene sequence arrangement is 5′-UTR-ORF1a-ORF1b-S-ORF3a-ORF3b-E-M-N-NS7-3′-UTR (Figure 1). TGEV ORF1a and ORF1b encode pp1a and pp1b, respectively, which can be hydrolyzed by papain-like protease and 3C-like protease to NSP1-16 (Van Reeth et al., 2002; Wang et al., 2018). Moreover, ORF3a/b and NS7 encode accessory proteins which are also involved in virus infection and virulence (Park et al., 2008). The TGEV S gene is about 4,344 bp and encodes S protein (1447aa), which has multiple functions, including inducing neutralizing antibodies, influencing host cell affinity, and determining virus activity (Gack et al., 2007). E protein of TGEV is a kind of membrane-associated small structural protein. A study has reported that 64-AYKNF-68 residues are the core sequences for binding E monoclonal antibodies (Yachdav et al., 2014). The M protein of TGEV consists of 263aa, is a kind of glycosylated protein that plays an important role in virus assembly. TGEV M protein is involved in inducing interferons (Sawicki et al., 2005). The N protein of TGEV is a conserved phosphorylated protein that binds to the genome to form an RNA complex. Because of its conserved nature, N is often used as an antigen for PEDV detection.

The SADS-CoV genome is approximately 27.2 kb in length and consists of 3′, 5′-UTR, and nine open reading frames, which are ORF1a, ORF1b, S, NS3a, E, M, N, NS7a, and NS7b in sequence (Pan et al., 2017; Xu et al., 2019a). SADS-CoV ORF1a encodes pp1a, which can be hydrolyzed to NSP 1–11. Moreover, ORF1b encodes pp1b, which can be hydrolyzed to NSP 12–16. NS3a, NS7a, and NS7b encode accessory proteins that affect viruses’ virulence (Xu et al., 2019a). The membrane protein interacts with the nucleoprotein of SADS-CoV during viral assembly and enhances viral transcription and assembly efficiency. The spike of SADS-CoV (1130aa) determines viral host range and tissue tropism. Notably, SADS-CoV S is highly homologous (95%) to bat α-coronavirus HKU2, which suggests that it may have the potential to spread across species (Yu et al., 2020).

The length of the PDCoV genome is approximately 25.4 kb, which is the smallest known coronavirus. PDCoV genome consists of 3′, 5′ UTR, and eight open reading frames, which are ORF1a, ORF1b, S, E, M, NS6, N, NS7a, and NS7 in order (Woo et al., 2010; Chen et al., 2015; Fang et al., 2017; Figure 1). PDCoV ORF1a and ORF1ab encode 1a polyprotein (3627aa) and 1ab polyprotein (6268aa), which can be hydrolyzed to NSP 2–16. It is widely accepted that PDCoV did not encode non-structural protein 1 (nsp1). These NSPs are associated with virus transcription, replication, and host immune response (Zhang, 2016). In addition, there are two ORFs between the M gene and N gene and within the N gene, which encode NS6, NS7a, and NS7. When PDCoV infects host cells, they are located in the endoplasmic reticulum (ER) and mitochondria, respectively (Fang et al., 2016; Choi and Lee, 2019). The structural analysis of PDCoV S protein showed that it was composed of S1 and S2 subunits. The N-terminal domain of PDCoV S1 recognizes carbohydrates as potential receptors, and the C-terminal domain of PDCoV S1 binds to receptors on the surface of mammalian cells (Shang et al., 2018). PDCoV N protein is located in the cytoplasm and nucleus of the host cell and participates in viral RNA synthesis by interacting with ribosomal subunits or nucleoproteins (Lee and Lee, 2015). In addition, PDCoV N protein is also involved in influencing the immune response of host cells (Likai et al., 2019).

## Innate Recognition of Porcine Enteric Coronavirus

Innate immunity is the first line of the host to defense against virus infection. The host cells recognize the invading pathogens through the interactions between PAMPs and host PRRs and induce the production of pro-inflammatory cytokines and interferons to elicit antiviral responses (Akira et al., 2006). When coronaviruses invade cells, PRRs, such as retinoic acid-inducible gene I (RIG-I)-like receptors (RLR) and Toll-like receptors (TLRs), are essential for the innate recognition of viral RNAs and are involved in the restriction of viral replication and dissemination. RLR, a family of cytoplasmic RNA helicases, including RIG-I, melanoma differentiation-associated gene 5 (MDA5), and Laboratory of Genetics and Physiology 2 (LGP2; Liu and Cao, 2016). Activation of RIG-I and MDA5 by double-stranded RNA (dsRNA) from coronaviruses leads to recruitment of the caspase recruitment domain (CARD)-containing adaptor protein mitochondrial antiviral signaling (MAVS) protein to activate TANK-binding kinase 1 (TBK1)/inhibitor-kb kinase ε (IKKε) kinases (Seth et al., 2005). Activated TBK1 and IKKε induce type I and type III IFNs production through phosphorylating interferon regulatory factors (IRFs; Meylan et al., 2006).

Other RNA sensors, TLR3, TLR7, and TLR8, located in the endosomal membrane, also recognize viral nucleotides, among which TLR3 recognizes dsRNA, TLR7 and TLR8 recognize ssRNA. The expression of TLR7 was significantly upregulated in PEDV-infected IPEC-J2 cells (Wang et al., 2020a). Cao et al. (2015) reported that PEDV infection induces nuclear factor-κB (NF-κB) activation through the TLR2, TLR3, and TLR9 pathways in porcine intestinal epithelial cells. PDCoV infection also significantly upregulates the mRNA transcription level of TLR3 and IFN-α *in vivo* (Xu et al., 2019b). In addition, TLR7 recognizes SARS-CoV, MERS-CoV, and MHV and induces IFN-a production in plasmacytoid dendritic cells (Cervantes-Barragan et al., 2007; Scheuplein et al., 2015). The receptors that sit on the surface of certain cells, especially TLR4, recognize MHV, SARS-CoV, SARS-CoV-2, and respiratory syncytial virus (Kurt-Jones et al., 2000; Khanolkar et al., 2009; Choudhury and Mukherjee, 2020). It has been reported that TLR4 also participates in PEDV infection-related pathogenesis (Huan et al., 2017). Once TLRs recognize the PAMPs of the virus, the factors NF-κB signaling will be activated to stimulate the production of pro-inflammatory cytokines and type I IFNs.

## Escape From Innate Immunity by Porcine Enteric Coronaviruses Structural Proteins

**Figure 2:**
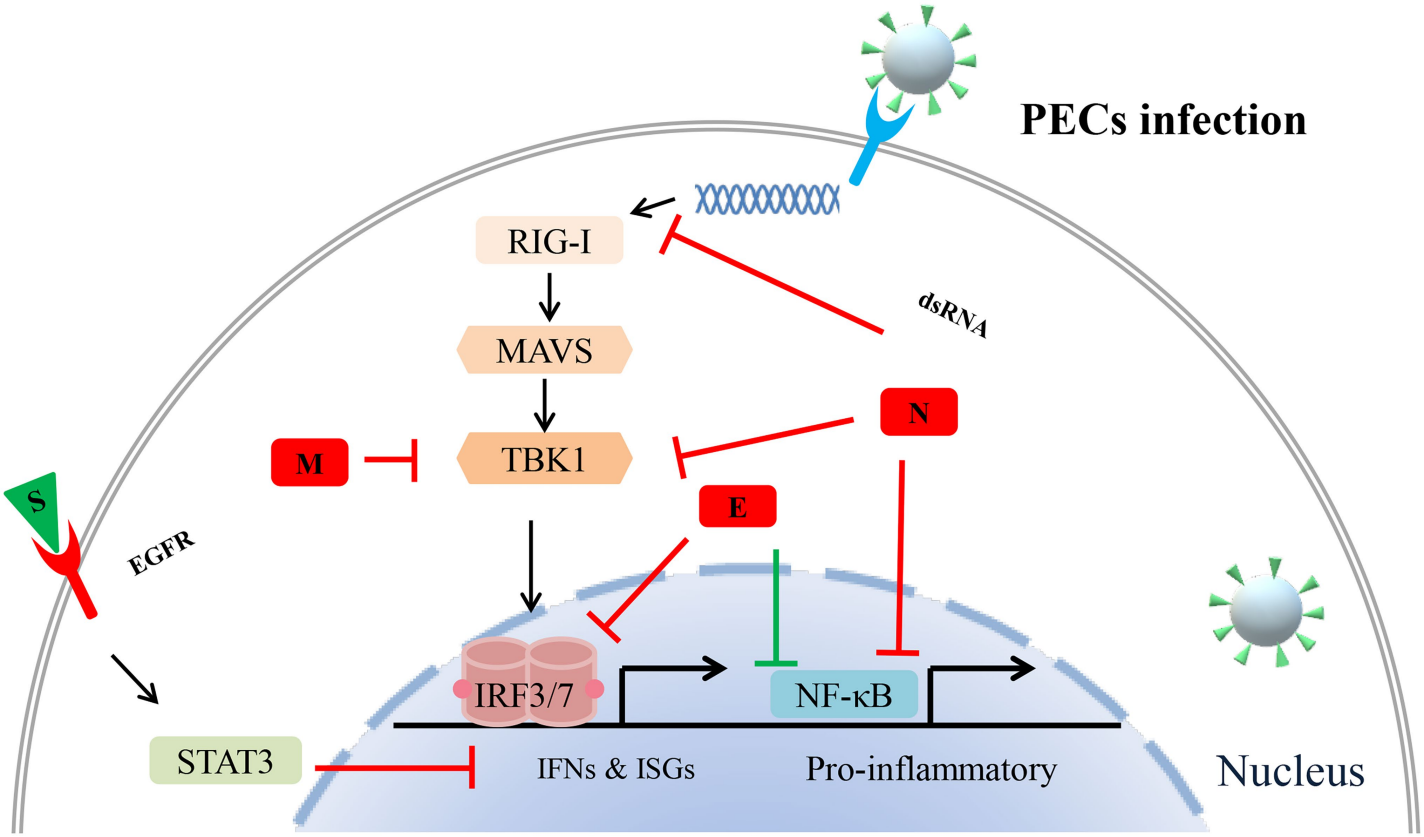
Potential mechanisms of porcine enteric coronavirus (PEC) structural proteins antagonize innate antiviral immune response. Different structural proteins of different porcine enteric coronaviruses used different strategies to antagonize the host’s immune responses. During PEC infection, interferons (IFNs) and pro-inflammatory are activated to fight against invading virus. It is noteworthy that STAT, retinoic acid-inducible gene I (RIG-I), and nuclear factor-κB (NF-κB) signalings are involved in this structural proteins-induced immune evasion. 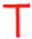 stands for negative regulation, 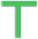 stands for positive regulation.

The structural proteins of porcine enteric coronaviruses consist of spike protein, envelope protein, membrane protein, and nucleocapsid protein. These proteins are essential components of viral structure and play important roles in fighting against the host’s immune responses (Figure 2). It is generally believed that the S protein of coronaviruses mainly plays a key role in the invasion of host cells and the induction of neutralizing antibodies (Song and Park, 2012; Li, 2015; Guan et al., 2020). However, a study has found that compared with other structural proteins and NSPs of PEDV, S protein has the strongest ability to induce apoptosis. Similarly, the S protein of TGEV can also strongly induce Vero-E6 cells apoptosis (Chen et al., 2018). Studies have suggested that some viruses actively induce apoptosis to promote the release of virus progeny and spread to neighboring cells for further invasion (Favreau et al., 2012; Lan et al., 2013). Thus, S protein probably helps these porcine enteric coronaviruses evade the host immune response by regulating apoptosis, although the exact mechanism is unclear. Moreover, another recent research has found that PEDV S protein directly interacts with epidermal growth factor receptor (EGFR) and activates EGFR downstream signal transduction, inhibiting IFN and exacerbating viral infection (Yang et al., 2018). Still, more evidence is needed to explore whether porcine enteric coronaviruses S protein is directly involved in viral immune escape or S protein mediates NSPs to realize virus immune escape.

The nucleocapsid protein is the most abundant protein of the known porcine enteric coronavirus components. It performs various functions, including viral genome transcription, translation, viral replication, and virus assembly (McBride et al., 2014). The PEDV N protein suppresses NF-κB nuclear translocation and further antagonizes Type II interferon production (Shan et al., 2018). Furthermore, the PEDV N protein targets TBK1 by direct interaction to inhibit IRF3 activation, further antagonizing type I interferon production. PDCoV N protein inhibits the activation of porcine IFN-β promoter by competing with dsRNA for porcine RIG-I binding (Chen et al., 2019). Moreover, the N-terminal region (1-246aa) of PDCoV N protein is the key part of interacting with porcine RIG-I (Likai et al., 2019). In addition, the N protein of PDCoV and SADS-CoV mediates K63-linked ubiquitination of porcine RIG-I, thereby, inhibit the host IFN-β production (Likai et al., 2019; Liu et al., 2021). The expression of the TGEV N gene promotes the accumulation of p53 and p21 and suppresses the expression of cyclin B1, cdc2, and cdk2. Meanwhile, TGEV N protein induces Bax mitochondria translocation and results in the activation of caspase-3, leading to apoptosis (Eleouet et al., 2000; Ding et al., 2014). In contrast, another study found that TGEV N protein, located in mitochondria, may contribute to mitophagy and suppress oxidative stress and apoptosis (Zhu et al., 2016). These different results may be due to their use of different cell lines for infection. These studies also suggested that the N proteins of different porcine enteric coronaviruses used different strategies to antagonize the host’s immune responses.

The member protein of porcine coronaviruses is a kind of transmembrane glycoprotein, which plays an important role in virion assembly, budding, and host immune regulation (Nguyen and Hogue, 1997; Riffault et al., 1997). Though the PEDV M protein affects cell cycle and interleukin 8 expressions, it does not induce ER stress and activation of NF-κB (Xu et al., 2015). Moreover, the PEDV M protein can form a complex with heat shock protein 70, affecting the host’s innate immune response and virus replication (Park et al., 2021). A recent study has identified 218 host cell proteins directly interacting with PDEV M protein. Moreover, these proteins were mainly associated with multiple biological processes such as immune response, apoptosis, and cell cycle (Wang et al., 2020b). In addition, some researches have reported that M proteins of TGEV and PDCoV help virus replication, which may be related to its regulation of IFN expression (Riffault et al., 1997; Gu et al., 2019; Li et al., 2019). So far, there are few studies on M proteins of porcine enteric coronavirus. M protein mediating porcine enteric coronavirus antagonism against host innate immune responses remains to be further studied.

The envelope protein is the smallest structural protein in porcine enteric coronavirus, involved in virus-host interactions. When the viruses invade the host cell, the E proteins are mainly located in the ER and play an essential role in virion assembly and budding (Xu et al., 2013a; Mora-Diaz et al., 2019). Based on its specific location in the cell, a study has found that PEDV E protein-induced ER stress and NF-κB activation upregulate the expression of IL-8 and Bcl-2. On the other hand, PEDV E protein directly interacts with IRF3 to inhibit its nuclear translocation, which further antagonizes interferon-β production (Zheng et al., 2021). The results of protein structure analysis suggest that porcine enteric coronavirus E protein may be involved in inducing humoral and cellular immunity during viral infection (Escors et al., 2001).

## Research Progress on Accessory Proteins of Porcine Enteric Coronaviruses Antagonizing Antiviral Innate Immune Responses

The porcine coronavirus accessory proteins are unique kinds of protein with special functions. Different numbers of accessory proteins are scattered in different porcine coronavirus genomes. Although they are unnecessary for virus proliferation, they play key roles in regulating innate immunity and viral pathogenicity (Fang et al., 2018; Wu et al., 2020b). PEDV has only one accessory protein, the ORF3. A study showed that ORF3 could suppress IFN-β and IRF3 promoter activities, but a detailed analysis of the certain mechanism is lacking (Zhang et al., 2016). Several studies have reported that PDEV ORF3 can interact with the host’s immune cells. PDEV ORF3 antagonizes the host’s antiviral innate immunity mainly by regulating NF-κB signaling pathway activity. PEDV ORF3 inhibits phosphorylation of IκBα and nuclear factor p65 and interfering p65 nuclear translocation, which in turn reduces the production of pro-inflammatory cytokines such as IL-6 and IL-8 (Wu et al., 2020b). Interestingly, ORF3 directly interacts with the IκB kinase β and upregulates the IκB kinase β-meditated NF-κB promoter activity. However, PEDV ORF3 suppresses the IκB kinase β-meditated IFN-β production (Kaewborisuth et al., 2020). Moreover, PEDV ORF3 induces ER stress *via* the PERK-eIF2α signaling pathway by upregulating the expression of GRP78, and then inducing autophagy, which benefits viral replication and affects the production of various inflammatory cytokines (Guo et al., 2017; Zou et al., 2019). In addition, conflicting studies have shown that proteins can inhibit or promote apoptosis, which is involved in viral replication and immune escape (Favreau et al., 2012; Si et al., 2020). Cells infected with Ns7-deletion mutant TGEV (TGEV-Δ7) showed an increased cytopathic effect by activation of caspase signaling. Further research found that the C-terminus of accessory protein 7 bound to protein phosphatase 1 catalytic subunit and regulated dephosphorylation of eukaryotic translation initiation factor 2 to counteract the host’s cell defenses (Cruz et al., 2011). Moreover, innate immunity genes such as IL-15, C–C motif chemokine 2/4/5, C–X–C motif chemokine 9/11, tumor necrosis factor, and IFN-β were upregulated during TGEV-Δ7 infection. *In vitro* and *in vivo* results suggested that the absence of TGEV accessory protein 7 increased innate immunity responses and acute tissue damage, which proved its antagonistic function from the opposite angle (Cruz et al., 2013). PDCoV accessory protein NS6 cannot prevent RIG-I, MDA5, and their downstream molecules from activating the IFN-β promoter. However, PDCoV NS6 can directly interact with the carboxyl terminus domain of RIG-I and the helicase and carboxyl terminus domains of MDA5 to inhibit dsRNA binding RIG-I/MDA5 and thus antagonize IFN-β production (Fang et al., 2018). PDCoV NS7a can also function as an IFN antagonist. Unlike NS6a, the NS7a inhibits RIG-I, MDA5, and their downstream molecules to activate the IFN-β promoter. Furthermore, NS7a can compete with TRAF3 and IRF3 for binding to IKK, thereby, reducing RLR-mediated IFN-β production. Moreover, the kinase and the scaffold dimerization domains of IKKε are key regions that can directly bond to NS7a (Fang et al., 2020). From those researches, the mechanisms by which different accessory proteins of porcine enteric coronaviruses suppress host antiviral innate immunity are different.

## Mechanism of Non-structural Proteins of Porcine Enteric Coronaviruses Antagonizing Innate Immune Response

Non-structural proteins are the earliest expression proteins essential for the virus replication process. They usually act as viruses evade, circumvent, or subvert the host innate immune system roles. During the process of porcine enteric coronavirus infection, NSP1, NSP3, NSP5, NSP15, and NSP16 have been observed to play additional roles in host immune-modulatory functions. Of 16 PEDV NSPs, NSP1, NSP3, NSP7, NSP14, NSP15, and NSP16 were found to inhibit the IFN-β and IRF3 promoter activities (Zhang et al., 2016). In addition, these porcine coronaviruses NSPs are also involved in downregulating the NF-κB activity (Zhang et al., 2017). In 2018, NSP1, NSP3, NSP5, NSP8, NSP14, NSP15, and NSP16 of PEDV were found to suppress type III IFN activities (Zhang et al., 2018). Here, we review the detailed mechanism of these non-structural proteins that antagonize interferon production.

Non-structural protein 1 is only characterized in alphacoronaviruses (α-CoVs) and betacoronaviruses (β-CoVs; Woo et al., 2010). Under the catalysis of the proteasome, the NSP1 of PEDV interrupted the enhanceosome assembly of IRF3 and CREB-binding protein (CBP) by degrading CBP to antagonize IFN-I production (Zhang et al., 2016). The CBP is the key molecular for the activated IRF3 to induce the transcription of IFN-I genes. After IRF3 phosphorylation and dimerization into the nucleus, IRF3 interacts with CBP to form the IRF3-CBP complex. And then, the complex binds to the positive regulatory domain (PRD) I–IV regions of the IFN-β promoter to assemble the enhanceosome with NF-κB and other factors to turn on the transcription of IFN-I genes (Honda and Taniguchi, 2006; Dragan et al., 2007; Panne et al., 2007). Another target gene for NSP1 to inhibit innate immunity is IRF1. IRF1 is the key adaptor protein for type III IFNs production. PEDV NSP1 blocked the nuclear translocation of IRF1 and reduced the number of peroxisomes to suppress IRF1-induced type III IFNs (Zhang et al., 2018). PEDV inhibited both NF-κB and pro-inflammatory cytokines production in porcine epithelial cells. Zhang et al. (2017) found that NSP1 was the most effective NF-κB antagonist among all proteins of PEDV. Moreover, NSP1 suppressed the phosphorylation and degradation of IκBα and blocked the p65 activation (Zhang et al., 2017). It is worth pointing out that the conserved residues of NSP1 were crucial to suppress IRF1-mediated IFN-λ and NF-κB mediated IFN-I and pro-inflammatory cytokines (Zhang et al., 2017, 2018). In addition, Shen et al. (2020) found that seven representative α-CoVs: SADS-CoV, PEDV, HCoV-229E, human NL63 CoV (HCoV-NL63), FIPV, TGEV, and PRCV NSP1s could significantly inhibit the phosphorylation of STAT1-S727 and interfere with the effect of IFN-I. The multiple functions of NSP1 to inhibit innate immune responses through different mechanisms suggest that it is one of the key molecules of porcine coronaviruses to escape innate immunity. Drugs targeting NSP1 conserved sites are likely to prevent and control these viruses.

NSP3 is the largest protein encoded by the porcine coronavirus genome and contains two domains of papain-like protease (PLP1 and PLP2). PLP2 has deubiquitinase (DUB) activity that recognizes and processes K-48 and K-63 linked polyubiquitin chains. Ubiquitin modification is a key mechanism to regulate the activity and stability of the antiviral innate immune. In recent years, several viral DUBs have been found to antagonize IFN-I production by deubiquitination of key host factors, such as the lead protease (Lbpro) of the foot-and-mouth disease virus (FMDV) and the NSP2 of Porcine Reproductive and Respiratory syndrome virus (PRRSV; Sun et al., 2010; Wang et al., 2011). Moreover, the DUB activity is conserved in all members of the arterivirus family. Both arteri- and nairovirus DUBs inhibit RIG-I mediated innate immune signaling (van Kasteren et al., 2012). The PLP2 of HCoV-NL63 and PLPs of SARS-CoV also antagonize IFN induction through disruption of STING dimer and deubiquitination of RIG-I (Chen et al., 2007; Clementz et al., 2010; Sun et al., 2012a). Accordingly, PEDV PLP2 strongly inhibits RIG-I- and STING-activated IFN expression by deubiquitination and co-immunoprecipitating with RIG-I and STING (Xing et al., 2013).

CoVs NSP5 and NSP3 genes encode 3C-like protease (3CLpro) and papain-like proteinase, respectively. These two proteinases can degrade the polyprotein into various non-structural proteins, which further facilitate virus replication. It has been found that many viruses’ 3C protease (3Cpro) antagonizes innate immune signaling pathways dependent on its protease activity. For example, encephalomyocarditis virus (EMCV) 3C protease cleaved TANK and disrupted the TANK-TBK1-IKKε-IRF3 complex, inhibiting IRF3 phosphorylation and IFN-I production (Huang et al., 2017). Coxsackievirus B 3C protease cleaves MAVS and TRIF to attenuate IFN-I and apoptotic signaling (Mukherjee et al., 2011). Enterovirus 71 3C protein induces TRIF cleavage to inhibit TLR-mediated antiviral responses (Lei et al., 2011). Similar to the Hepatitis A virus and FMDV 3Cpro, PEDV and PDCoV 3C-like proteases cleave NEMO to impair induction of IFN-β (Wang et al., 2012, 2014a, 2016). The cleave site of NEMO has been identified at Gln231 both in PEDV and PDCoV, suggesting NEMO may be a common target for coronaviruses (Wang et al., 2016; Zhu et al., 2017a). However, it cannot exclude the possibility that other non-active site residues of their NSP5 are also involved. Soon afterward, other target molecules of PDCoV NSP5 inhibit IFN-I signaling was revealed. Like NS5 protein of dengue virus (DENV), Zika virus (ZIKV) and the hepatitis C virus (HCV), PDCoV NSP5 target the JAK–STAT pathway to antagonize IFN-I signaling (Lin et al., 2006; Ashour et al., 2009; Grant et al., 2016). In PDCoV-infected cells, NSP5 cleaved STAT2 at glutamine 685 (Q685) and Q758As to impair ISGs induction (Zhu et al., 2017b). As NSP5 is involved in the cleavage of the viral polyprotein, the inhibitors target its 3C-like protease domain that can suppress porcine enteric coronavirus infection, such as quercetin, GC376 (Zhou et al., 2019; Ye et al., 2020).

NSP15 is identified as a component of the coronavirus replication complex, which has endoribonuclease (EndoU) activity. The role of EndoU was revealed, which showed that EndoU mediates the evasion of viral double-stranded RNA recognition by host sensors in macrophages. In previous studies, SARS-CoV NSP15 was identified as an inhibitor of MAVS-mediated apoptotic responses (Lei et al., 2009). MHV and HCoV-229E NSP15 efficiently prevent simultaneous activation of host cells dsRNA sensors, such as MDA5, OAS, and PKR (Kindler et al., 2017). A study has reported that the EndoU activity of PEDV NSP15 is not required for virus replication. Still, PEDV NSP15 is important for suppressing the type I and type III IFN response in epithelial cells and macrophages. NSP15 facilitates virus replication, shedding, and pathogenesis *in vivo* (Deng et al., 2019). With the study forward, the mechanism of PEDV NSP15 inhibits the host’s IFN response was found. PEDV NSP15 can directly degrade the mRNA of TBK1 and IRF3 dependent on its EndoU activity to suppress the production of IFN and ISGs, antagonizing the host innate response to facilitate its replication (Wu et al., 2020a). PDCoV NSP15 is also an IFN antagonist. However, PDCoV NSP15 disrupts the phosphorylation and nuclear translocation of the NF-κB p65 subunit but does not antagonize the activation of transcription factor IRF3. Moreover, PDCoV NSP15 inhibits IFN-β production independent of EndoU activity (Liu et al., 2019).

NSP16 is one of the RNA modification enzymes involved in forming cap structures in PEDV (Chen et al., 2011). Compared with NSP14, which is another methyltransferase in PEDV, NSP16 is a more efficient regulator in the antagonist of innate immunity. Mechanistically, NSP16 downregulates the activities of RIG-I and MDA5 mediated IFN-β and ISRE dependent on the KDKE tetrad. Moreover, NSP10 enhanced the inhibitory effect of NSP16 on IFN-β (Shi et al., 2019). However, whether NSP16 of PDCoV and SADS-CoV antagonizes interferon production is still unknown.

**Figure 3:**
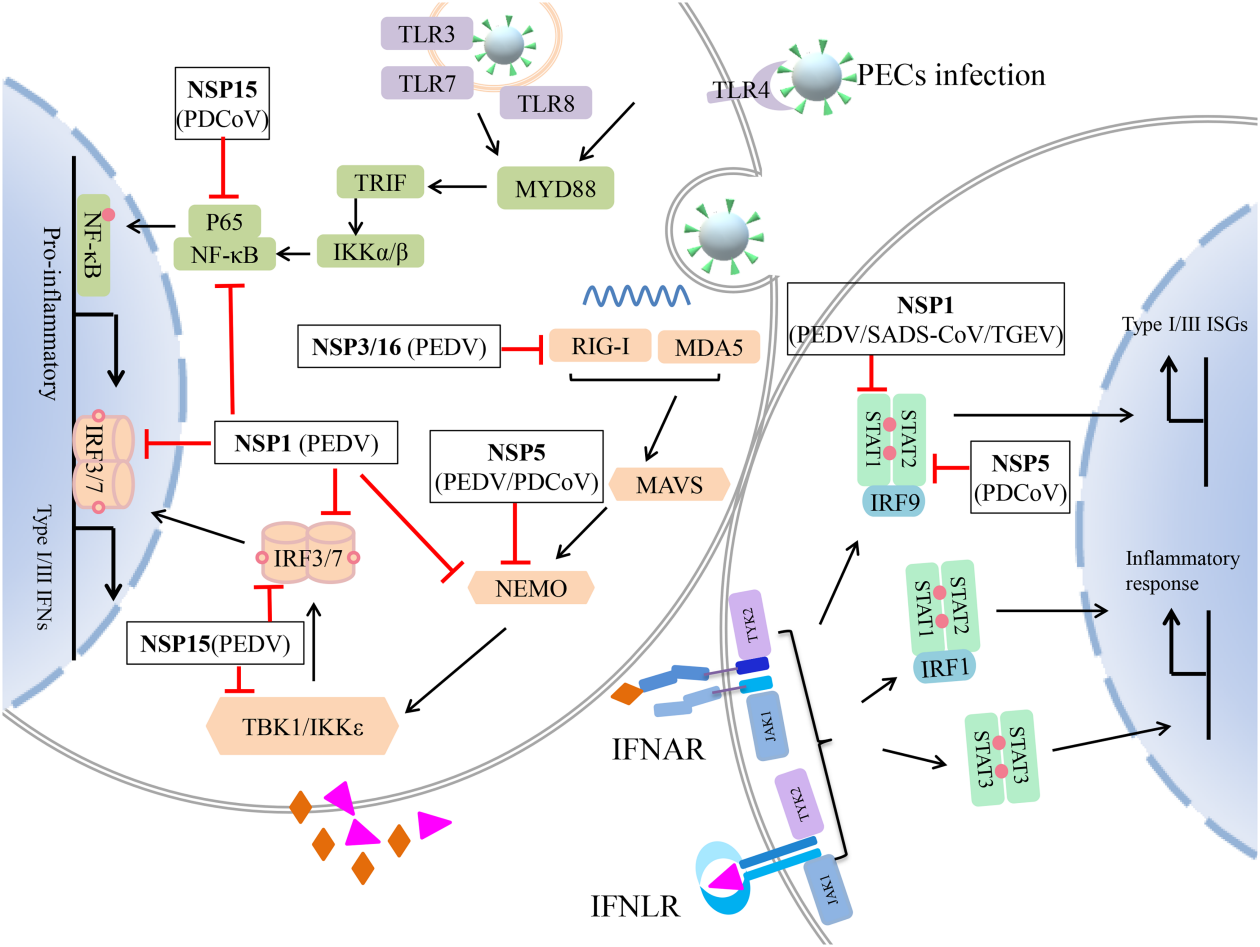
Non-structural protein (NSP) of porcine enteric coronavirus (PEC) antagonizes innate immune response. Retinoic acid-inducible gene I (RIG-I), melanoma differentiation-associated gene 5 (MDA5), and Toll-like receptors (TLRs) recognize the invading virus and induce pro-inflammatory cytokines, type I/III interferons (IFNs) by nuclear factor-κB (NF-κB) and (RIG-I)-like receptors (RLRs) signaling pathway, respectively. Extracellular Type I and III IFNs recognized by IFN-I receptor (IFNAR) and type III IFN receptor (IFNLR) to phosphorylate JAK1 and TYK2. And then, STAT1/2 is recruited and phosphorylated to form three STAT complexes. STAT1 and STAT2 form heterodimers and recruit IRF9 or IRF1. These complexes enter the nucleus and induce type I and III ISGs, inflammatory cytokines production. NSPs of PEC antagonize various steps of this antiviral response. 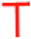 stands for negative regulation; 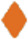 stands for type I IFN; and 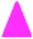 stands for type III IFN.

These studies suggest that the NSPs of porcine enteric coronaviruses antagonize the host’s innate immune responses by regulating IFN signaling pathways (Figure 3). Therefore, further study of the biological functions of NSPs will help us elucidate the pathogenesis of coronaviruses and possibly provide new targets for developing antiviral vaccines and drugs.

## Conclusion and Perspectives

The host’s innate immune response protects itself from most pathogenic microorganisms, but some viruses have evolved strategies to antagonize innate immune responses. Coronaviruses are the largest positive-sense RNA viruses that exist widely in nature and are highly genetically variable. This review summarizes how porcine enteric coronaviruses evade the host’s innate immune responses. First, since IFN is the most important regulator of the antiviral innate immunity, these viruses typically inhibit IFN production by various means, including inhibition of RIG-I/TLR signaling and inhibiting dsRNA bind to RIG-I/MDA5 or directly downregulates IFN promoter activity. Second, porcine enteric coronaviruses also attenuate inflammatory response by targeting the NF-κB signaling pathway. In addition, some porcine enteric coronaviruses can regulate apoptosis and evade ISGs to promote virus replication.

Also, some cellular physiological processes, such as autophagy, endoplasmic reticulum stress, programmed cell death, are probably involved in the evasion of the innate immune response of these viruses (Lin et al., 2020; Sun et al., 2021; Wei et al., 2021). Some viruses use autophagy to evade the host immune response and hide in the autophagosome to accumulate RNA and proteins (Sun et al., 2012b; Liu et al., 2016). A recent study has reported that PEDV infection induced autophagy, which promotes virus replication. Moreover, NSP6 and ORF3 of the virus are two of the important inducers of autophagy. Further study showed that PI3K/Akt/mTOR pathway is the key signal of PEDV NSP6-induced autophagy (Lin et al., 2020). During porcine coronavirus infection, some of the viral proteins are located in the endoplasmic reticulum of host cells. E protein, N protein, and ORF3 of PEDV can all induce ER stress *via* PERK and IRE1 signaling and then upregulate inflammatory factors (Xu et al., 2013a,b; Sun et al., 2021). In addition, PEDV-induced ER stress facilitates autophagy (Zou et al., 2019). Furthermore, TGEV infection in porcine intestinal epithelial cells can induce IL-1β release and pyroptosis, dependent on the expression and assembly of the NOD-like receptor protein 3. The above evidence highlights the importance of investigating virus-host interactions to elucidate viral immune evasion. Some viruses would like to alter the intracellular environment to ensure their survival. Exploring these programs will help us further understand how porcine enteric coronaviruses evade innate immune responses and also provide us with new ideas for developing antiviral vaccines and drugs.

## Author Contributions

KZ, SL, SD, JL, JZ, and SW conceived and wrote the manuscript. KZ and SW prepared the figure. All authors approved the final version of this review and agreed to be accountable for the content of the work.

## Funding

The authors thank the following funding sources: Independent Research and Development Projects of Maoming Laboratory (2021ZZ003), the Special Fund for Scientific Innovation Strategy-Construction of High-Level Academy of Agriculture Science (R2021PY–QF006, R2019YJ–YB2005, R2019YJ–YB2004, and R2019YJ–YB3002), and the Science and Technology Planning Project of Guangzhou (202103000096).

## Conflict of Interest

The authors declare that the research was conducted in the absence of any commercial or financial relationships that could be construed as a potential conflict of interest.

## Publisher’s Note

All claims expressed in this article are solely those of the authors and do not necessarily represent those of their affiliated organizations, or those of the publisher, the editors and the reviewers. Any product that may be evaluated in this article, or claim that may be made by its manufacturer, is not guaranteed or endorsed by the publisher.
